# Lassa Fever 2016 Outbreak in Plateau State, Nigeria—The Changing Epidemiology and Clinical Presentation

**DOI:** 10.3389/fpubh.2018.00232

**Published:** 2018-08-29

**Authors:** Nathan Y. Shehu, Simji S. Gomerep, Samson E. Isa, Kelly O. Iraoyah, Johnson Mafuka, Nandom Bitrus, Matthias C. Dachom, John E. Ogwuche, Asukwo E. Onukak, Kenneth I. Onyedibe, Ephraim Ogbaini-Emovon, Daniel Z. Egah, Elizabeth J. Mateer, Slobodan Paessler

**Affiliations:** ^1^Infectious Disease Unit, Department of Medicine, Jos University Teaching Hospital, Jos, Nigeria; ^2^Irrua Specialist Teaching Hospital, Irrua, Nigeria; ^3^Department of Pathology and Institute for Human Infections and Immunity, University of Texas Medical Branch, Galveston, TX, United States

**Keywords:** lassa, fever, epidemiology, clinical presentation, Nigeria

## Abstract

Lassa fever (LF) outbreaks in Nigeria mostly occur in rural areas and during the dry season, peaking between December through February. Fever is a cardinal presenting feature among the myriad manifestations of LF. Thirty four patients with clinical diagnosis of LF were analyzed. However, only 11 (32%) LASV infections were confirmed by RT-PCR. The 2016 LF outbreak showed a preferential urban occurrence and a high case fatality. Fever (≥38°C) was not detected in over a fourth of the patients at the time of examination. Bleeding diathesis was the most common presentation while abdominal pain and headache were present in more than half of the confirmed cases. Changes in the geographical distribution and clinical presentation may have implications for disease control efforts and the risk of transmission, both locally and internationally. In order to guide interventions, public health authorities should be aware that the epidemic patterns may be changing.

## Introduction

Lassa virus (LASV, *Arenaviridae*) is the causative agent of Lassa fever (LF), which is a systemic infectious disease that can lead to acute hemorrhage as well as neurological disorders (1). Due to international travel, LF poses a significant risk to both visitors to endemic countries and to populations in non-endemic regions.

Lassa fever was brought to the consciousness of the medical community world-wide when a nurse became infected in 1969 while working in a small missionary hospital at Lassa a town in North East Nigeria (2). Lassa fever is widely distributed across West Africa where its reservoir, *Mastomys natalensis*, is commonly found. In addition to Nigeria, reports of LF from Guinea and Sierra Leone have been published (3–7). Lassa fever is endemic in West Africa placing 2 million persons per annum at risk of becoming infected, with 5,000–10,000 fatalities annually (1). It also causes outbreaks in Nigeria yearly with peaks in December through February (2).

Most infections with LASV in Africa are asymptomatic, mild or subclinical, but case fatality in hospitalized patients can be as high as 15–25% (3, 7). Clinical manifestations are variable, and often nonspecific as many of those are seen in other febrile infectious diseases in endemic areas. The incubation period of LF is between 3 and 21 days. Ribavirin, a nucleoside analog, is the only drug with significant therapeutic benefit for patients when administered within 6 days of onset of illness (8, 9). In a recent LF outbreak between January and April of 2016 in Nigeria, a total of 268 cases (suspected, probable and confirmed) and 147 deaths were reported in 23 of the 36 states. From those, 164 cases were confirmed in the laboratory by RT-PCR (10). A clinical and epidemiologic study done in Plateau state, Nigeria, between 2012 and 2013 showed that 100% of Lassa fever patients were from Rural/sub-urban residence, and 100% presented with fever, 83.3% with hemorrhage, 66.7% with cough, 33.3% with sore throat, 33.3% with proteinuria, and 16.7% with retrosternal chest pain (11). We set out to characterize the 2016 outbreak in Jos in order to determine potential change in epidemiology and to determine clinical correlates of LF.

## Methodology

### Study design

This retrospective study was carried out between January and August 2016, at the Jos University Teaching Hospital (JUTH), Nigeria. It is a referral center for most states of the north central zone of Nigeria. The study was approved by the Ethics Committee at JUTH, reference number JUTH/DCS/ADM/1271/XXV/13 dated 22nd February, 2017. Demographic, clinical features, laboratory test and outcomes were obtained from patient records and entered into a proforma. The study population consisted of patients with suspected LF based on WHO case definition of suspected LF as follows: Presence of fever of >38°C for <3 weeks, absence of clear focus of infection and lack of response to broad spectrum antibiotics or anti-malarials for 48 h. Additionally, presence of any two major signs (bleeding from orifice(s), facial/neck swelling, conjunctivitis, spontaneous abortion, petechial rashes, persistent hypotension, elevated liver enzymes, known contact with LF patients or one major criterion with any two minor criteria (headache, vomiting, cough, sore throat, diffuse abdominal pain, retrosternal pain, profound weakness, myalgia/arthralgia and proteinuria) (12).

### Laboratory diagnosis

Blood samples from all suspected patients were collected using EDTA bottle and transported in cold chain to the Lassa Fever Reference Laboratory at Irrua Specialist Teaching Hospital, Edo State, Nigeria. The blood was centrifuged and virus RNA was purified and immediately used for Reversed -Transcriptase PCR (RT-PCR). The Lassa virus RT-PCR targeting the GPC gene was performed using QIAGEN OneStep RT-PCR Kit reagents as described (13, 14). A primers set: S 36^+^ (5′- ACC GGG GAT CCT AGG CAT TT-3′) and LVS_339-d^−^ (5′-GTT CTT TGT GCA GGA MAG GGG CAT KGT CAT-3′), which targets well conserved specific region in the Glycoprotein precursor protein (GPC) of the S RNA segment of the Lassa virus was used. PCR products were separated and detected in a 1.5% agarose gel containing ethidium bromide and visualized by UV light. As a positive control, inactivated culture supernatant of cells infected with Lassa virus strain CSF was used.

### Data analysis

Statistical analysis was done using Epi Info™ 7.0.9.34. Categorical variables were compared using Chi square test or Fisher's exact test. Continuous variables were expressed as mean ± standard deviation or as median with range. Means were compared by student's t test and *p* < 0.05 was considered significant.

## Results

**Figure 1:**
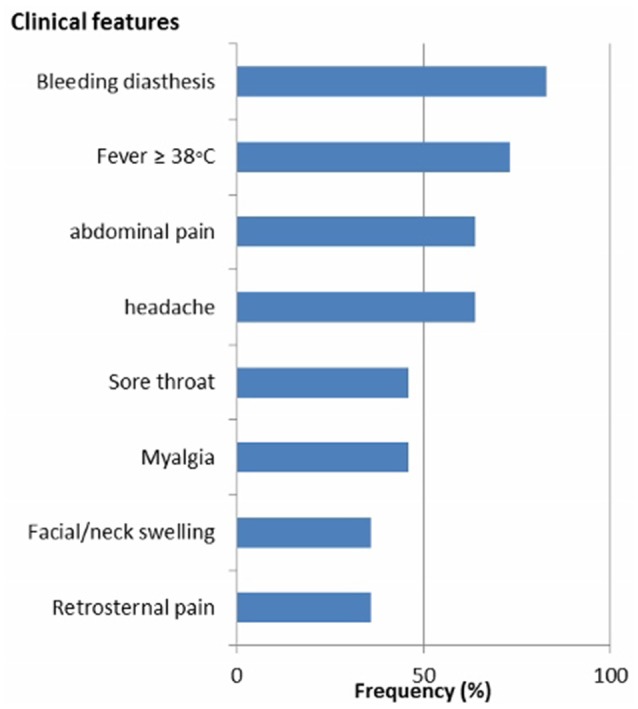
Frequency of the clinical features of confirmed Lassa fever patients in Jos, North central Nigeria.

Clinical and epidemiological data of 34 patients with suspected LF were analyzed. Their mean age (SD) was 31 ± 11 years and 18 (53%) of patients were females. All Socio-demographic characteristcs are presented in Table 1. There was no significant difference in the sociodemographic profile of suspected and confirmed cases (Table 1). Eleven (32%) cases were confirmed by RT-PCR. The case fatality for the confirmed cases was 36%. Among the confirmed cases 8 (73%) resided in urban residential areas while 3 (27%) were from rural areas. The frequency of the clinical presentation of confirmed cases is presented in Figure 1. Bleeding diasthesis, abdominal pain, fever (>38°C), headache, sore throat, myalgia, and retrosternal pain were not found to be associated with confirmed LF except facial swelling Table 2. The mortality in the confirmed LF and negative were (4) 36% and [6)] 26% respectively, *p*-value of 0.69.

**Table 1:** Socio-demographic characteristics of suspected Lassa fever patients.

**Variable**	**Positive PCR *n* = 11**	**Negative PCR *n* = 23**	***P*-value**
**Mean age (SD) (years)**	32 (7)	31 (13)	0.75
**Age group {*****n*** **(%)}**			
15–25	2 (18)	11 (48)	
26–35	5 (46)	5 (22)	
>35	4 (36)	7 (30)	0.2
**Sex**			
Female	6 (55)	12 (52)	0.9
Males	5 (45)	11 (48)	
**Occupation**			
Business	4 (36)	3 (13)	
Farmers	2 (18)	3 (13)	
Health care workers	2 (18)	1 (4)	
Students	1 (9)	5 (22)	
Office workers	1 (9)	5 (22)	
Unemployed	1 (9)	5 (22)	0.40

**Table 2:** Clinical and epidemiological factors associated with confirmed vs. negative Lassa fever cases.

**Variables**	**Positive *n*(%) *n* = 11**	**Negative *n*(%) *n* = 23**	^*^P-value
Bleeding diasthesis	9 (82)	13 (57)	0.29
Facial/neck swelling	4 (36)	1 (4)	0.03
Headache	7 (64)	12 (52)	0.72
Myalgia	5 (46)	5 (22)	0.23
Fever ≥38°C	8 (73)	16 (70)	1.00
Urban residence	8 (73)	19 (83)	0.66
Retrosternal pain	4 (36)	5 (22)	0.43
Sore throat	5 (46)	9 (39)	1.00
Abdominal pain	7 (64)	13 (57)	1.00
Duration on admission [mean(*SD*)](Days)	9 (4)	7 (4)	0.33

* *Fishers exact*.

## Discussion

We present a possible change in epidemiology and clinical presentation detected during the 2016 LF outbreak in Jos, North central Nigeria. The main finding of this study is an epidemiologic drift in the occurrence of LF from rural to predominantly urban residential areas (73%). This is a marked deviation from an earlier study done in the same study area in 2012 that found rural frequency of 100% (11). A study in Guinea found a higher prevalence of Lassa virus IgG [12.9% [10.8–15.0%]] in rural areas than in urban areas [10.0% [8.1–11.9%]] (15). This may be due to greater abundance of the natural host, *M. natalensis*, in rural settings. The predominant occurrence of LF in urban areas from our study may be due to relative abundance of the natural host in this setting due to poor sanitation and overcrowding. Poor sanitation and crowded areas have been shown to predispose individuals to LF (16). Additionally, McCormick et al. demonstrated that *M. natalensis* constituted 50–60% of the rodents captured in houses but only 10–20% of those captured in surrounding agriculture and bush areas (3). This suggests that houses are the most important locations for transmission of LASV.

Our observed case fatality of 36% is fairly similar to 33% reported in Benin republic 2016 LF outbreak (17). But it is higher than the 15–25% case fatality previously reported in hospitalized patients (7). This is likely due to their reporting of case fatality among both suspected and confirmed cases. However, the fatality rate in this study is lower than the case fatality rate we found in a similar study in 2012 (83%) (11). Although, there is an improved access to ribavirin in 2016, patients still presented late to the hospital. There is low index of suspiction among clinicians as LF is often considered only after patients have been presumptively treated for malaria and typhoid fever without improvement. Also, ignorance of the disease and superstitious believe among the local population may also contribute to late presentation. For example, in this outbreak, a relation was accused to have been responsible for the deaths through witchcraft of a woman and her two children who died of LF. Therefore, there is an urgent need to create more awareness of LF in the community and to train and retrain clinicians and other healthcare workers on the clinical detection and management of Lassa Fever, particularly on the need for early referral of all suspected cases to designated treatment centers in-country. Early clinical presentation and referral to specialist center for rapid treatment. This would go a long way in reducing the mortality in the future.

The common clinical features were bleeding diasthesis, abdominal pain, fever >38°C, headache and sore throat with frequencies of over 40%. Our results are very similar to the study by Ehichioya et al. that found fever, abdominal pain, headache and sore throat to be present in more than 40% of patients (18). Nevertheless, fever was recorded in 100% of their cohorts whereas we found 73% in our cohorts. The reason for this difference might be caused by our higher cut-off value for fever of >38°C. Additionally, the frequent use of antipyretics (fever suppressive drugs) by patients prior to presentation at the hospital may also account for the relative lower prevalence of fever among our cohort. Nevertheless, fever of >38°C is the most significant alert clinical feature for early case detection of LF, which is of pivotal importance for patient triage and care. Missing fever in suspected patients may delay LF diagnosis and treatment, which could negatively impact clinical outcome and increase the chances for secondary transmission. Sudden onset sensorineural hearing loss (SNHL) has been reported to occur in one-third of LF survivors during the convalence phase of disease and constitutes a neglected public health and social burden (19). Due to the reported high prevalence of LF-induced SNHL, it is important to better understand the clinical presentation for the future patients. Recently, an animal model that mimics the pathology of LF-induced SNHL in humans has been described. This new model will hopefully enable scientists to better characterize the mechanim(s) of LF-induced SNHL and lead to better diagnostic tools (20).

The frequent occurrence of abdominal pain, fever, headache, sore throat in sub-Saharan Africa calls for more predictive clinical features and improved diagnostics. Only facial swelling was found to be significantly associated with confirmed LF cases. In a similar study in Sierra Leone, the two important clinical features that suggest diagnosis of LF are abnormal bleeding and swollen face and/or neck (21). Facial swelling is one of the major diagnostic criteria of LF (12). Facial and neck swelling can easily be identified in a patient. Why facial swelling occurs in LF is not fully understood. It may be due to renal impairment, intravenous fluid administration or some yet to be identified mechanism or combination of all. Aside from local conditions affecting the head and neck, hypersensitivity reaction and thyroid disease, facial swelling is not usually reported in other severe bacterial and viral infections. However, McCormick and colleagues found that the best predictor for LF detection is the combination of fever, pharyngitis, retrosternal pain and proteinuria (7). Due to the highly variable clinical presentation of LF, a high index of suspicion is needed especially when there is relevant epidemiology and constellation of clinical features. This would lead to early diagnosis and reduction of mortality. Lassa fever is difficult to distinguish from other viral haemorrhagic fevers and other diseases that cause fever, including malaria, typhoid fever, leptospirosis, and yellow fever (19). These febrile illnesses along with other severe bacterial infections at terminal stages, may present with bleeding diasthesis making it difficult to clinically distinguish any of these from with LF. Additionally, LF has variable and non-specific clinical features making clinical diagnosis difficult (20). Therefore, laboratory confirmatory diagnosis is critical in diagnosis. The bleeding diasthesis and mortality seen in our cohorts who were are Lassa PCR negative may be due to severe bacterial infections, yellow fever, dengue or other viral haemorrhagic fevers. Comparing the mortality rates of LF positive and negative, the proportion of mortality among the confirmed LF patients were 10% higher than those of negative cases. However, this is not statistically significant. This implies that other severe febrile illness have comparable fatalities with LF. There is need to consider a broader laboratory diagnostic platform that would in parallel screen for other viral haemorrhagic fevers and other eatologies of acute febrile illnesses in endemic area and more research is needed to identify other possible unknown etiologies.

Lassa fever in Nigeria is known to predominantly occur in the dry season, which usually lasts from December to March (11). The reasons for this periodicity may be due to increase movement of the natural host into houses and therefore easier contact with to humans. However, we also found the occurrence of LF in August indicating that infection potentially can occur at any time of the year. Similarly, in Guinea, as in other West African countries, LF is known to occur throughout the year but is more common in the dry season due to higher numbers of *M. natalensis* in homes due to restricted food supply (22).

The main limitation of the study is the smaller sample size, which may limit the ability for generalized conclusions and inability to document preceding history of travel during the incubation period before presentation to the hospital.

In conclusion, this small- scale-study suggests that among all the suspected LF cases seen during the 2016 LF outbreak in North Central Nigeria, there was no difference in the socio-demographic profile and clinical manifestation of confirmed cases and negative cases, except for neck swelling. An emerging epidemiologic trend was noted with more cases occurring in urban than rural areas and with increased case fatality. There is need to scale-up awareness campaign and surveillance; heighten index of suspicion and promote early diagnosis and referral of all suspected cases in order to reduce morbidity and mortality.

## Author contributions

NS conceived the study and design, acquired the data, performed data analysis, interpretation and drafted the manuscript. SG conceived the study and design, acquired the data and reviewed the manuscript. SI conceptualized the design, acquired the data and reviewed the manuscript. KI, JM, NB, MD, JO, AO, and KO conceptualized the design and reviewed the manuscript. EO-E methodology and manuscript review. DE supervised infection control and reviewed the manuscript. EM and SP analyzed, interpreted and made a critical review of the manuscript.

### Conflict of interest statement

The authors declare that the research was conducted in the absence of any commercial or financial relationships that could be construed as a potential conflict of interest.
